# Hepatitis B Vaccination in People Living with HIV: Bridging the Immunological Gap

**DOI:** 10.3390/vaccines14070623

**Published:** 2026-07-16

**Authors:** Christelle Radi, Jana Abu Faraj, Jad Idriss, Daniel J. Gromer, Diane Saint-Victor, Christiane S. Eberhardt, Nadine Rouphael, Suha Kalash

**Affiliations:** 1 Devision of Infectious Diseases, Department of Medicine, Emory University School of Medicine, Atlanta, GA 30322, USA; 2 Division of Infectious Diseases, Department of Pediatrics, Children’s Healthcare of Atlanta, Emory University School of Medicine, Atlanta, GA 30322, USA; 3 Center for Vaccinology, University Hospitals of Geneva, 1211 Geneva, Switzerland; 4 Center for Vaccinology, University of Geneva, 1211 Geneva, Switzerland

**Keywords:** hepatitis B virus, HIV, hepatitis B vaccination, people living with HIV, vaccine immunogenicity, seroprotection, vaccine nonresponse, HepB-CpG, occult hepatitis B infection, isolated anti-HBc, intensified vaccination strategies, vaccine implementation

## Abstract

Hepatitis B virus (HBV) infection remains a major driver of liver-related morbidity and mortality among people living with HIV (PLWH), yet vaccine-induced protection is frequently suboptimal. HIV-associated immune dysfunction, including CD4^+^ T-cell depletion, altered antigen presentation, impaired T follicular helper cell support, and B-cell dysregulation, reduces seroprotection after standard recombinant HBV vaccines and may limit durability of antibody responses. Vaccine response is further influenced by HIV viral suppression, age, comorbidities, prior vaccine history, and baseline HBV serologic status, including isolated hepatitis B core antibody (anti-HBc) and occult HBV infection (OBI) considerations. Although antiretroviral therapy (ART) improves vaccine responsiveness, many PLWH fail to achieve protective hepatitis B surface antibody (anti-HBs) titers (≥10 mIU/mL) after conventional schedules, or experience antibody waning over time. Current guidelines recommend HBV vaccination for all susceptible PLWH with post-vaccination serologic testing and revaccination for nonresponders. Persistent implementation barriers, including incomplete series, vaccine hesitancy, stigma, and logistical constraints, continue to limit real-world impact. Emerging clinical trial data support CpG-adjuvanted HBV vaccines (HepB-CpG/Heplisav-B) and intensified dosing and schedules (double-dose or four-dose regimens) to improve seroprotection and generate higher peak anti-HBs titers, which may enhance durability. This review synthesizes guideline recommendations, immunologic mechanisms of hyporesponsiveness, predictors of vaccine response, and practical strategies to optimize HBV vaccination in PLWH.

## 1. Introduction

Hepatitis B virus (HBV) imposes a major burden on global health. The World Health Organization (WHO) estimates that 254 million people were living with chronic HBV infection in 2022, with approximately 1.2 million new infections occurring each year. In the same year, HBV was associated with an estimated 1.1 million deaths [1]. Among people living with HIV (PLWH), approximately 5–20% are co-infected with HBV [2], with marked variation across different regions. In endemic areas, HBV transmission is mainly through perinatal exposure or early childhood contact with infected blood. In adults, transmission is mainly via sexual contact, injection drug use, and unsafe medical practices [3]. HIV and HBV share common transmission routes [4]. HBV infection poses a significant risk for PLWH as HIV/HBV coinfection is associated with more rapid progression of liver disease, including cirrhosis, hepatocellular carcinoma, and liver-related mortality, compared with HBV monoinfection [5,6]. HIV impairs both innate and adaptive immune responses, and co-infection is associated with elevated HBV DNA levels, delayed hepatitis B antigen clearance, and increased risk of chronic infection and liver disease progression [7,8].

Occult HBV infection (OBI), generally defined as persistence of replication-competent HBV DNA in the liver, with or without detectable serum HBV DNA, despite negative hepatitis B surface antigen (HbsAg), adds an additional layer of complexity in PLWH. In this population, OBI and isolated hepatitis B core antibody (anti-HBc) serologic patterns may reflect prior HBV exposure with incomplete immune control, low-level viral persistence, or immune escape. HIV-associated immune dysfunction, particularly when HBV-active antiretroviral therapy (ART) is absent, withdrawn, or interrupted, may increase the risk of HBV reactivation [9,10,11,12,13].

Despite the availability of effective vaccines for people without HIV, eliciting robust and durable immunity against HBV in PLWH remains challenging. Because PLWH are at higher risk of HBV acquisition and less likely to mount durable vaccine responses, optimizing vaccine selection, dosing schedules, and follow-up testing is central to HBV prevention in HIV care [14].

In this review, we outline the challenges of HBV vaccination in PLWH, focusing on the magnitude and durability of the antibody response, seroconversion rates, and key predictors of suboptimal immunogenicity. We also explore strategies to improve protection in this population, including dose escalation, additional booster doses, alternative delivery routes, and use of novel adjuvants. Finally, we discuss real-world hurdles to effective implementation of vaccination strategies.

## 2. Methodology

This article was developed as a narrative, not systematic, review of the literature on hepatitis B virus vaccination in people living with HIV. Relevant articles, clinical trials, systematic reviews, meta-analyses, guideline documents, and public health reports were identified through searches of PubMed, Google Scholar, guideline websites, and public health agency resources, including the CDC, NIH, and WHO. Search terms included combinations of “hepatitis B vaccine,” “HBV vaccination,” “HIV,” “people living with HIV,” “vaccine response,” “seroprotection,” “anti-HBs,” “nonresponse,” “revaccination,” “double-dose,” “four-dose schedule,” “intradermal vaccination,” “Heplisav-B,” “HepB-CpG,” “CpG adjuvant,” “isolated anti-HBc,” “occult hepatitis B infection,” “implementation barriers,” and “stigma.”

Articles were selected based on their relevance to HBV vaccine immunogenicity, predictors of vaccine response, durability of antibody responses, guideline recommendations, alternative vaccination strategies, OBI/anti-HBc management, and implementation challenges in people living with HIV. Priority was given to clinical trials, systematic reviews, meta-analyses, major society guidelines, and recent studies addressing CpG-adjuvanted vaccines or intensified vaccine schedules. Because the objective was to synthesize clinically relevant evidence rather than to perform a systematic review, formal protocol registration, duplicate screening, risk-of-bias assessment, and independent searches of Web of Science, Embase, and the Cochrane Library were not undertaken. The evidence was synthesized narratively to summarize current recommendations, immunologic mechanisms of hyporesponsiveness, practical vaccination strategies, and barriers to implementation.

## 3. Guidelines for Hepatitis B Vaccination in People Living with HIV

Several leading health authorities, including the Centers for Disease Control and Prevention (CDC), the National Institutes of Health (NIH), and the World Health Organization (WHO), have issued guidance relevant to HBV vaccination in PLWH.

These recommendations differ in scope: CDC guidance includes broad U.S. population-level vaccination and screening recommendations that also apply to PLWH, NIH guidance provides HIV-specific recommendations for adults and adolescents living with HIV, and WHO guidance is framed for global and national immunization programs. Across these frameworks, susceptible PLWH without chronic HBV should be vaccinated after baseline HBV serologic assessment.

In this review, a “protective response” or “seroprotection” refers to hepatitis B surface antibody (anti-HBs) ≥10 mIU/mL measured approximately 1–2 months after completion of the vaccine series. This threshold is used to document vaccine response, but in PLWH the magnitude of the peak response is also important because higher titers, particularly ≥100 mIU/mL in selected contexts, are associated with more durable antibody persistence.

Interpretation of baseline serology is particularly important in PLWH with isolated anti-HBc, a common and clinically nuanced pattern defined by negative HBsAg, positive total anti-HBc, and absent or low anti-HBs. It is reported in approximately 7–19% of PLWH and represents a heterogeneous clinical entity [7]. It may reflect prior resolved HBV infection with subsequent waning of anti-HBs, false-positive anti-HBc results, occult HBV infection with low-level viremia, or less commonly the window phase of acute infection [7]. In PLWH, isolated anti-HBc is more frequently associated with prior exposure and immune dysregulation and may carry a small but clinically relevant risk of occult HBV infection (OBI) or viral reactivation [3,9,10,11,12,13].

Accordingly, management requires a nuanced approach. Current HIV-specific guidance recommends vaccination for PLWH who have isolated anti-HBc followed by anti-HBs testing [15]. A single-dose “challenge” strategy is intended to distinguish a memory/anamnestic response from apparent susceptibility or a weak primary response [7]. If anti-HBs rises to ≥100 mIU/mL after the challenge dose, no further vaccination is generally needed; if the titer remains <100 mIU/mL, a complete series is recommended because many individuals with isolated anti-HBc behave immunologically as primary responders rather than as persons with durable memory. If vaccination is refused, follow-up serology is not feasible, ART lacks HBV activity, a tenofovir-sparing switch is planned, or additional immunosuppression is anticipated, HBV DNA testing and ALT monitoring should be considered to evaluate for OBI or reactivation risk; vaccination alone should not be used to prevent reactivation of established OBI [12,13].

Currently available hepatitis B vaccines include recombinant alum-adjuvanted formulations such as Engerix-B and Recombivax HB, typically administered as a three-dose series (0, 1, and 6 months), as well as the CpG-adjuvanted vaccine Heplisav-B, given as a two-dose series over one month. Combination vaccines such as Twinrix (hepatitis A and B) are also available. A trivalent HBV vaccine, PreHevbrio, was previously available but has been withdrawn from the market, though not for safety nor for effectiveness reasons [16,17].

The CDC recommends HBV vaccination for all adults aged 19–59 years and for those ≥60 years with risk factors, including HIV, whereas the NIH and WHO provide specific guidance for PLWH. Pediatric PLWH follow pediatric HIV guidance and current routine or catch-up HBV schedules; Table 1 focuses on recommendations most relevant to adolescent and adult PLWH rather than reproducing all pediatric schedules. Regarding pregnancy, direct safety data specifically in pregnant PLWH remain limited. Recommendations to vaccinate susceptible pregnant individuals with HIV are largely extrapolated from the extensive pregnancy experience with recombinant HBV vaccines in the general pregnant population and from the benefit of preventing HBV in PLWH. Alum-adjuvanted recombinant hepatitis B vaccines have extensive safety experience in pregnancy. For HepB-CpG (Heplisav-B), pregnancy data were previously limited; however, newer post-marketing data have not shown an increased risk of adverse pregnancy outcomes compared with alum-adjuvanted hepatitis B vaccines [18].

Key similarities and distinctions are summarized in Table 1.

## 4. Immunologic Determinants of HBV Vaccine Hyporesponsiveness in PLWH

HIV infection reduces the immunogenicity of HBV vaccination [28]. This reduced vaccine responsiveness is largely attributed to HIV-associated immune dysregulation, particularly impairments in T-cell as well as antigen-presenting cell (APC) function, T-cell help, germinal center function, B-cell memory and the inflammatory milieu needed for durable humoral immunity.

T-cell dysfunction: CD4^+^ T helper cells, especially T follicular helper cells (Tfh), play a central role in supporting germinal center formation, class-switch recombination, affinity maturation, and the production of high-affinity anti-HBs antibodies. HIV infection leads to both quantitative CD4^+^ T-cell depletion and qualitative dysfunction including impaired cytokine production and altered costimulatory signaling, which reduce the ability of T cells to provide help to HbsAg-specific B cells [29,30]. As a result, many PLWH fail to mount protective antibody responses following HBV vaccination [31]. Higher CD4^+^ T-cell counts at the time of vaccination are strongly associated with improved HBV seroconversion rates, whereas lower CD4^+^ T-cell counts, persistent immune activation and incomplete CD4+ T-cell recovery are linked to reduced seroconversion and vaccine non-responsiveness [2,30].

APC impairment and innate immune activation: HIV also impairs the function of key APC subsets, including dendritic cells and monocytes. These cells exhibit reduced expression of co-stimulatory molecules and diminished capacity for antigen processing and presentation, leading to suboptimal T-cell priming and impaired vaccine-induced immunity [32,33]. Chronic immune activation, microbial translocation, and dysregulated interferon signaling may further blunt vaccine responsiveness by creating an inflammatory environment that is activated but inefficient for coordinated adaptive immune priming.

B-cell dysregulation: Moreover, HIV leads to marked B-cell dysregulation, characterized by chronic polyclonal B-cell activation, functional exhaustion, and an expansion of atypical B cells that overexpress inhibitory receptors [34,35]. HIV infection also drives the depletion of memory B-cell pools, further compromising the capacity for long-term immunity [36]. This HIV-associated B-cell dysfunction is associated with impaired vaccine responses. Notably, higher frequencies of resting memory B cells have been correlated with improved anti-HBs antibody responses following HBV vaccination in PLWH [37]. More broadly, studies in healthy HBV-vaccinated adults show that stronger anti-HBs responses correlate with higher HBsAg-specific memory B-cell frequencies. In one study, high responders also had greater IL-13 production, suggesting a Th2-associated contribution to humoral responses after conventional HBV vaccination [38].

These mechanisms help explain why strategies that increase antigenic stimulation or enhance innate immune activation, such as double-dose or four-dose recombinant schedules and TLR9 agonist CpG-adjuvanted vaccines, can improve seroprotection in PLWH. They also support routine PVST and documentation of the numeric anti-HBs titer, because a low-level response may indicate inadequate germinal-center and memory B-cell formation even when the conventional seroprotection threshold is reached.

## 5. Factors Associated with Optimal HBV Vaccine Immune Responses in PLWH

Understanding factors associated with HBV vaccine response in PLWH is essential to elucidate the mechanisms of impaired immunogenicity and guide more effective vaccination strategies. These factors can be grouped into HIV-related factors, host and comorbidity factors, and vaccine-related factors.

HIV-related factors: Effective ART partially reverses HIV-induced immune dysfunction by suppressing viral replication and improving CD4^+^ T-cell counts, both of which are key determinants of vaccine responsiveness. A prospective study of PLWH in China confirmed that both low CD4 counts and elevated HIV viral loads independently predicted poor vaccine responses [39]. Several studies have also shown that HIV RNA suppression to undetectable levels is independently associated with improved vaccine-induced antibody responses, supporting the role of viral control in restoring humoral immunity [30,40].

PLWH who are on ART with suppressed viral loads and CD4^+^ T-cell counts >350 cells/μL demonstrate significantly higher seroconversion rates to standard HBV vaccination [17]. For example, in a Ugandan cohort of PLWH with a median CD4^+^ count of 426 cells/μL, 92% achieved protective anti-HBs titers following a standard three-dose recombinant alum-adjuvanted hepatitis B vaccine (Euvax); among those receiving ART, most were virologically suppressed [31]. Earlier ART initiation may be important because complete restoration of B-cell function may require ART during the early stages of HIV infection [36].

Host and comorbidity factors: In addition to CD4 count and HIV viral load, older age is a consistently recognized predictor of lower HBV vaccine response in both the general population and PLWH [23,30,40]. Other factors associated with reduced response across studies include male sex in some cohorts, smoking, obesity or high body mass index, diabetes, chronic kidney disease or dialysis, chronic liver disease, HCV coinfection, lower CD4 nadir, prior vaccine nonresponse, and incomplete series adherence [23,30,39,40]. The strength and consistency of these associations vary by study design, vaccine product, and baseline immune status, but they are useful for identifying patients who may benefit from an optimize-first strategy.

Vaccine-related factors: Product, antigen dose, number of doses, schedule completion, and timing of PVST all influence the measured response. HBV vaccination should not be deferred solely to await CD4^+^ recovery in susceptible PLWH with ongoing exposure risk [17]; instead, PVST can identify nonresponders who may benefit from revaccination or an intensified strategy after immune reconstitution on ART. In addition, vaccine-induced anti-HBs responses in PLWH may wane more rapidly, warranting closer follow-up and consideration of boosters in those with ongoing risk [41].

## 6. Optimizing Strategies for HBV Vaccination in PLWH

Despite the availability of both alum-adjuvanted and CpG-adjuvanted hepatitis B vaccines, standard vaccination regimens often result in suboptimal immunogenicity in PLWH, particularly in individuals with low CD4 counts, unsuppressed HIV RNA, prior nonresponse, or ongoing immune dysfunction [30]. When anti-HBs concentrations fall below protective thresholds, booster doses are recommended, and in some cases, revaccination with alternative dosing strategies or adjuvanted formulations may be required to restore and maintain durable immunity against HBV [7,17,41]. The conventional HBV vaccination schedule for adults consists of three doses given at 0, 1, and 6 months (Recombivax HB [Merck&Co., Inc., Rahway, NJ, USA] and Engerix-B [GlaxoSmithKline, London, UK]). PLWH often exhibit a diminished response to this schedule, with seroconversion rates ranging from 34% to 88.6% [40]. This contrasts with the general population where the standard HBV vaccination induces protective antibody concentrations in over 90–95% of healthy adult individuals [42]. Even among those who initially achieve seroconversion, antibody titers may decline more rapidly in PLWH compared with immunocompetent individuals, leading to earlier loss of protective immunity [36]. Current recommendations therefore emphasize monitoring of hepatitis B surface antibody (anti-HBs) levels in PLWH [7,17,41]. Unlike immunocompetent individuals, in whom immune memory may persist despite waning antibody levels, PLWH may be less likely to maintain long-term protection after loss of detectable anti-HBs levels because of HIV-associated immune dysfunction and impaired memory B-cell responses [41]. For a patient with documented prior completion of a vaccine series and a documented protective post-series anti-HBs titer who later has anti-HBs < 10 mIU/mL with negative HBsAg and anti-HBc, the pattern is best considered waning immunity rather than primary nonresponse. In PLWH with ongoing exposure risk, especially if not receiving HBV-active ART, a single booster or challenge dose with repeat anti-HBs testing is a reasonable first step; if there is no response, full revaccination with an optimized regimen should be completed according to local guidance.

To enhance vaccine responsiveness, higher-dose and extended vaccination regimens have been evaluated. The 2021 meta-analysis by Tian et al., which included 17 studies and 1821 PLWH reported that a double-dose regimen (40 µg) improved seroconversion rates to 75.2%, compared with 65.5% with the standard 20 µg dose. Similarly, a four-dose schedule achieved an 89.7% response rate, substantially outperforming the conventional three-dose schedule (63.3%) [30]. Similarly, the 2020 systematic review and meta-analysis by Lee et al., with nine trials and 970 PLWH, confirmed that double-dose vaccination enhances the magnitude of immune responses and is associated with higher response rates at both early and long-term follow-up [43]. These findings support the use of intensified dosing strategies in PLWH, particularly in individuals with incomplete immune reconstitution despite ART.

Accelerated HBV vaccination regimens have been investigated to provide more rapid initial protection for PLWH, particularly those at increased risk of HBV infection, such as men who have sex with men and people who inject drugs. They are also relevant for individuals requiring urgent immunity, including travelers to HBV-endemic regions and healthcare workers with potential occupational exposure [17]. In a randomized controlled trial of PLWH (aged ≥ 18 years) who were hepatitis B-naïve, adherence to an accelerated regimen (0-, 1-, and 3-week regimen) was significantly higher compared to the standard schedule (91.8% versus 82.7% *p* ≤ 0.001). However, the accelerated schedule did not improve seroprotection rates relative to the standard regimen. Notably, among individuals with CD4^+^ T-cell counts of 200–500 cells/mm^3^, the serologic response, as measured by anti-HBs antibody levels, was 20.8% lower with the accelerated regimen [44]. Thus, accelerated schedules may improve completion or speed early dosing but should not be assumed to improve immunogenicity, and PVST remains essential.

The route of administration may also influence HBV vaccine immunogenicity in PLWH. While the intramuscular (IM) route is standard, the intradermal (ID) route has been explored as an alternative, given the high density of APCs in the skin. In a randomized controlled trial of PLWH, three regimens were compared: a standard-dose IM schedule (20 mcg at weeks 0, 4, and 24), a double-dose IM schedule (40 mcg at weeks 0, 4, 8, and 24), and a low-dose ID schedule (4 µg at weeks 0, 4, 8, and 24). At week 28, serological response rates were 65% for the standard IM group, 82% for the IM double-dose group, and 77% for the ID group, indicating that both intensified IM and ID regimens enhanced immunogenicity compared to standard dosing and route [45]. However, findings across studies are mixed. One randomized controlled trial comparing ID and IM routes found a lower response with the ID route (Geometric Mean Titer of 157 vs 459, respectively, *p* < 0.0001) [46]. Another study involving PLWH who did not respond to the standard HBV vaccination schedule evaluated a single booster dose administered either via ID or IM routes and found that both approaches improved serological responses, with no significant difference in response rates between the two delivery methods (47.6% in the ID group and 50% in the IM group) [47]. Discrepant findings may reflect differences in age, baseline immune status, CD4 thresholds, prior nonresponse, antigen dose (e.g., 4 mcg ID versus 20–40 mcg IM), number of doses, technique-dependent ID delivery, skin thickness, and whether outcomes emphasize response rate or geometric mean titer. Thus, ID vaccination is biologically plausible but remains less standardized than optimized IM strategies.

CpG-adjuvanted HBV vaccines provide an immunologic rationale for overcoming some HIV-associated vaccine hyporesponsiveness. CpG oligodeoxynucleotides activate Toll-like receptor 9 (TLR9), leading to plasmacytoid dendritic cell and B-cell activation, type I interferon secretion, stronger innate immune stimulation, and Th1-polarized responses that can augment antibody production and cellular immunity [48,49]. Among PLWH, data have shown that HepB-CpG elicits a stronger serologic response compared to conventional alum-adjuvanted vaccines. In a study involving PLWH with prior nonresponse to HBV vaccination, both the two-dose and three-dose regimens of HepB-CpG vaccine achieved significantly higher seroprotection rates than the standard three doses of HepB-alum vaccine [50]. Similarly, in a recent phase 3 trial of vaccine-naïve PLWH, administration of the HepB-CpG vaccine resulted in 100% of PLWH achieving seroprotective anti-HBs titers after three doses, with no serious safety concerns reported [49]. Notably, a randomized controlled trial demonstrated that the addition of CpG 7909 to HBV vaccination considerably increased seroprotection rates and antibody titers in PLWH. Anti-HBs titers were assessed every six months for up to five years. Participants who received the CpG 7909 adjuvant were more likely to achieve and maintain seroprotective antibody levels across all follow-up points [51]. These findings suggest that investigational CpG-adjuvanted strategies may represent a promising future direction for improving HBV vaccine responses in PLWH.

We summarize a practical workflow for screening, vaccination, PVST, and revaccination in Box 1.

Box 1 **Proposed pragmatic approach to HBV vaccination in PLWH [7,9,10,11,12,13,17,30,43,44,45,46,47].**

- (1) Screen and document baseline status before vaccinating.

○ Order baseline HBV serologies: The HBV triple panel—HBsAg, total anti-HBc, and anti-HBs. “Total anti-HBc” is used for baseline exposure assessment and anti-HBc IgM is reserved for suspected acute infection.
○ Document: Prior HBV vaccine history (product, doses, dates), prior anti-HBs results, current ART regimen (including whether it has HBV-active agents), planned ART switches and ongoing exposure risk.

- (2) Interpret serology and triage.

○ HBsAg positive: Evaluate/confirm chronic HBV (e.g., HBV DNA, ALT, staging as appropriate) and manage as HBV infection. Vaccination is not the pathway for the patient, but vaccinate/assess close contacts as indicated.
○ Immune: if anti-HBs ≥ 10 mIU/mL with HBsAg negative → document immunity (no routine revaccination needed). In individuals with ongoing high-risk exposure or substantial time since vaccination → consider periodic reassessment of anti-HBs levels according to local guidance and risk profile. If anti-HBc is also positive, consider this resolved prior to infection and assess reactivation risk before HBV-active ART interruption, nucleos(t)ide-sparing ART switches, or immunosuppression.
○ Susceptible: If HBsAg negative, anti-HBc negative, anti-HBs < 10 mIU/mL → proceed to vaccination. If a prior complete vaccine series and prior protective anti-HBs ≥ 10 mIU/mL are documented but current anti-HBs is <10 mIU/mL, treat as waning immunity rather than primary nonresponse; for ongoing-risk PLWH, consider a single booster/challenge dose followed by anti-HBs testing, and complete revaccination if no response.
○ Isolated anti-HBc (HBsAg negative, anti-HBc positive, anti-HBs < 10): Consider a single “challenge” dose followed by anti-HBs testing 1–2 months later. If anti-HBs is ≥100 mIU/mL, no further vaccine is generally needed; if anti-HBs remains <100 mIU/mL, complete a full series and repeat anti-HBs testing. If follow-up cannot be assured or OBI/reactivation risk is clinically important, consider HBV DNA and ALT assessment.
○ Suspected OBI or anti-HBc-positive patient at reactivation risk: Vaccination may help prevent new infection or assess immune memory, but it should not be used as a strategy to prevent reactivation of established OBI. Manage with HBV DNA/ALT assessment, HBV-active ART when indicated, and monitoring according to local guidance.

- (3) Vaccinate using an “optimize-first” mindset.

○ Use a preferred product/schedule per local guidance, with a bias toward strategies shown to improve immunogenicity in PLWH (e.g., CpG-adjuvanted vaccine and/or intensified dosing schedules).
○ Consider an intensified strategy upfront (higher-dose and/or additional doses) for individuals with predictors of poor response (e.g., low CD4 count, unsuppressed HIV RNA, older age, prior nonresponse, significant comorbidities, or anticipated poor follow-up), when feasible.

- (4) Verify response (PVST):

○ Check anti-HBs 4–8 weeks (up to 16 weeks if delayed follow-up) after the final dose.
○ Define response as anti-HBs ≥ 10 mIU/mL and document the numeric titer (not just “positive/negative”). In PLWH, higher post-vaccination titers (e.g., >100 mIU/mL) may be associated with more durable protection and stronger memory B-cell responses, whereas lower-level responses may wane more rapidly over time.

- (5) If anti-HBs < 10 mIU/mL:

○ Confirm series completion and timing of PVST.
○ If modifiable HIV factors exist, optimize (e.g., achieve viral suppression, immune reconstitution) and then revaccinate rather than abandoning vaccination.
○ Revaccinate using an optimized strategy (e.g., switch vaccine platform/adjuvant or use a higher-dose/more-dose regimen), then repeat PVST.

- (6) Address durability in those with ongoing risk.

○ For patients with continued exposure risk and/or advanced immune dysfunction, consider periodic anti-HBs monitoring and booster dosing if titers wane below protective thresholds, particularly if not receiving HBV-active ART.

- (7) Build implementation into workflow.

○ Schedule follow-up doses and PVST at the first vaccine visit, use EHR prompts/standing orders, and prioritize simplified regimens when completion is a concern.

## 7. Implementation Challenges

Despite evidence supporting intensified HBV vaccination strategies in PLWH, real-world implementation faces multiple challenges. In a cohort of 502 PLWH in specialty HIV care, 59% were missing one or more CDC-recommended vaccinations [52]. Structural and logistical barriers limit vaccine delivery, particularly in resource-limited settings. Cold chain requirements and supply constraints remain major obstacles to timely hepatitis B birth-dose implementation [53]. Multiple studies show that storing hepatitis B vaccine outside the cold chain can be feasible and can improve timely administration when properly implemented [54,55,56,57]. At the global level, disparities in HBV vaccine delivery highlight broader implementation challenges. Although the WHO estimates that 84% of individuals complete the three-dose HBV vaccine series, timely administration of the birth dose remains suboptimal, with only 45% of newborns receiving it worldwide and the lowest coverage observed in the WHO African Region [58]. These gaps reflect systemic barriers in vaccine delivery that may also affect implementation of adult vaccination strategies in PLWH [58].

Stigma represents an additional and often underrecognized barrier to HBV vaccination in PLWH. HIV-related stigma remains a major barrier to prevention and care among PLWH. The conceptual and empirical literature has linked stigma to reduced healthcare engagement, poorer treatment adherence, and adverse health outcomes. In healthcare settings specifically, HIV-related stigma has also been associated with lower uptake of HIV prevention and care services, including testing, PrEP, linkage to care, retention in care, and medication adherence [59,60].

HBV-related stigma is often driven by misconceptions about transmission, fear of social exclusion, and concerns about disclosure. Prior reviews have shown that HBV stigma can negatively affect disclosure, help-seeking, screening, linkage to care, and access to treatment [61,62]. In PLWH, these overlapping stigmas can compound one another and contribute to missed vaccination opportunities and incomplete vaccine series.

Healthcare provider knowledge and attitudes represent an additional barrier. In a survey of 314 healthcare workers in Northern Vietnam, the median knowledge score regarding HBV prevention and management was 59.5%, equivalent to correctly answering 25 of 42 questions on transmission, vaccine safety, disease outcomes, and preventive measures. Notably, 38.8% of respondents lacked confidence in HBV vaccine safety and 48.2% reported unsafe needle-handling practices. Physicians and healthcare workers who had received training within the prior two years scored significantly higher [63]. Similarly, a survey of 528 healthcare workers in Cambodia found that while 60% were aware of HBV transmission mechanisms, only 32% had themselves received HBV vaccination [64].

Several implementation-focused interventions have been shown to improve HBV vaccination uptake. Integration of vaccination into routine HIV care, use of simplified or accelerated schedules, and delivery through community-based or harm-reduction settings can increase series completion, particularly in high-risk populations [28]. In addition, provider education and training were associated with improved vaccine delivery and reduced missed opportunities for vaccination [63].

These findings underscore the importance of comprehensive education and training for both patients and providers. Targeted interventions that reduce stigma, simplify vaccination schedules, and strengthen healthcare worker knowledge are essential to improving HBV vaccine uptake and achieving better health outcomes among vulnerable populations.

## 8. Conclusions

HBV vaccination is a core preventive intervention in HIV care, yet vaccine-induced protection remains less reliable in PLWH. HIV disrupts multiple steps required for durable humoral immunity, including antigen presentation, CD4^+^ T-cell help, and memory B-cell generation. Suppressive ART and higher CD4 counts improve responsiveness, but they do not fully eliminate hyporesponsiveness or the risk of more rapid antibody waning, underscoring that vaccination in PLWH is not “one size fits all.” Accordingly, vaccine selection and schedule intensity should be paired with routine post-vaccination serologic testing to confirm protection and to trigger timely, dose-optimized revaccination for nonresponders.

Evidence summarized in this review supports a practical shift toward strategies that increase the likelihood of high-titer responses, such as CpG-adjuvanted formulations and intensified dosing schedules, because higher peak anti-HBs titers may translate into more durable protection. Finally, the impact of any immunogenicity advantage will be blunted without effective delivery systems: series completion, PVST, and clear revaccination pathways must be operationalized in HIV clinics, alongside interventions that address hesitancy, stigma, and logistical barriers. Based on these considerations, a pragmatic, stepwise approach to HBV vaccination in PLWH is proposed to guide clinical decision-making and implementation in routine care (Box 1).

## Figures and Tables

**Table 1:** Selected CDC, NIH, and WHO guidance relevant to Hepatitis B vaccination in people living with HIV [1,7,14,16,19,20,21,22,23,24,25,26,27].

Guideline Component	CDC	NIH	WHO **
Screening Before Vaccination	Population-level CDC guidance recommends universal hepatitis B virus (HBV) screening for adults at least once using a triple panel: Hepatitis B surface antigen (HbsAg), hepatitis B surface antibody (anti-HBs), and total hepatitis B core antibody (anti-HBc).	HIV-specific guidance recommends screening PLWH for HBV infection and immunity, including HBsAg, anti-HBs, and total anti-HBc.	WHO guidance supports HBV testing and linkage to care according to national context, with PLWH prioritized in many HBV/HIV programs where testing is available.
Vaccination Recommendation and Age Scope	Adults 19–59 years: vaccination recommended, including people living with HIV (PLWH). Adults ≥ 60 years with risk factors, including HIV, should be vaccinated. Persons ≤ 18 years should follow current age-appropriate CDC routine/catch-up schedules; pediatric schedules are not fully reproduced in this adult-focused table.	Vaccinate all susceptible PLWH. Heplisav-B is preferred for previously unvaccinated adults/adolescents with HIV when available; alternative intensified recombinant vaccine schedules may be used.	Universal infant vaccination is central to WHO guidance. Adolescent/adult vaccination, including for PLWH, is implemented according to national policy, vaccine availability, and risk-group strategies.
Vaccination Schedule	Standard 3-dose recombinant series at 0, 1, and 6 months; adult 2-dose HepB-CpG may be used when age-appropriate. Pediatric schedules vary by age and product.	Preferred: Heplisav-B at 0 and 4 weeks for vaccine-naive PLWH when available. Alternatives: double-dose recombinant vaccine series at 0, 1, and 6 months (e.g., Engerix-B 40 mcg or Recombivax HB 20 mcg) or other locally recommended schedules.	Schedules are program-defined and commonly use recombinant multi-dose vaccine series; national programs determine adult risk-group schedules.
Post-Vaccination Serologic Testing (PVST)	PVST 1–2 months after the final vaccine dose is recommended for groups for whom clinical management depends on knowing immune status, including PLWH.	PVST 1–2 months after the final vaccine dose is recommended for PLWH; response is anti-HBs ≥ 10 mIU/mL.	PVST is not routinely recommended for all vaccinees in population programs; it is used in selected groups/settings (e.g., infants born to HBsAg-positive persons or local high-risk protocols).
Management of Non-Responders	If PVST is indicated and anti-HBs remains <10 mIU/mL after a complete series, repeat a complete vaccine series and repeat PVST.	Revaccinate nonresponders using an optimized strategy, preferably HepB-CpG if the prior series used alum-adjuvanted vaccine, or intensified recombinant schedules when HepB-CpG is unavailable; repeat PVST.	Because routine PVST is not universal in WHO population programs, there is no single global nonresponder algorithm for all vaccinees. If nonresponse is identified through local PVST protocols, revaccination is guided by national policy and risk context.
Vaccination During Pregnancy	Vaccinate pregnant patients who need HepB vaccination. Alum-adjuvanted recombinant vaccines have the longest pregnancy experience; current CDC guidance also permits other adult HepB products when indicated.	Do not defer indicated HBV vaccination solely because of pregnancy in susceptible PLWH. Direct PLWH-specific pregnancy safety data are limited; recommendations rely largely on general pregnancy safety data and benefit-risk considerations.	WHO emphasizes prevention of perinatal and early-childhood HBV through birth-dose/infant vaccination programs; adult vaccination during pregnancy follows national policy and product availability.

The CDC column summarizes U.S. population-level guidance relevant to PLWH; the NIH column is HIV-specific; and the WHO column reflects global/programmatic guidance that may not specify the same PLWH-specific adult product hierarchy. The table focuses on PLWH and does not reproduce all routine pediatric, birth-dose, or catch-up schedules. The NIH follows U.S. FDA-approved vaccines. Commonly recommended HBV vaccines for PLWH in NIH guidance include Engerix-B, Recombivax HB, Heplisav-B, and Twinrix when age- and indication-appropriate. ** WHO also endorses recombinant HBV vaccines derived from yeast (e.g., Saccharomyces cerevisiae); common WHO-prequalified vaccines include Euvax-B, Shanvac-B, Engerix-B, Recombivax HB, and Biovac-B.

## Data Availability

No new data were created or analyzed in this study. Data sharing is not applicable to this article.

## Abbreviations

The following abbreviations are used in this manuscript:

PLWH	People living with HIV
HBV	Hepatitis B virus
ART	Antiretroviral therapy
Anti-HBs	Hepatitis B surface antibody
Anti-HBc	Hepatitis B core antibody
HBsAg	Hepatitis B surface antigen
CD4^+^ T-cell	CD4^+^ T lymphocyte
T_fh_	T follicular helper cell
APC	Antigen-presenting cell
PVST	Post-vaccination serologic testing
CDC	Centers for Disease Control and Prevention
WHO	World Health Organization
NIH	National Institutes of Health
OBI	Occult hepatitis B Infection
